# Diffusion and self-assembly of *C*_60_ molecules on monolayer graphyne sheets

**DOI:** 10.1038/srep21910

**Published:** 2016-02-25

**Authors:** Masoumeh Ozmaian, Arman Fathizadeh, Morteza Jalalvand, Mohammad Reza Ejtehadi, S. Mehdi Vaez Allaei

**Affiliations:** 1 Institute for Nanoscience and Nanotechnology, Sharif University of Technology, Tehran, Iran; 2 Department of physics, University of Tehran, Tehran 14395-547, Iran; 3 School of physics, Institute for research in fundamental sciences (IPM), Tehran, Iran; 4 Department of Physics, Sharif University of Technology, P.O. Box 11155-9161, Tehran, Iran; 5 Center of Excellence in Complex Systems and Condensed Matter (CSCM), Sharif University of Technology, Tehran 1458889694, Iran

## Abstract

The motion of a fullerene (*C*_60_) on 5 different types of graphyne is studied by all-atom molecular dynamics simulations and compared with former studies on the motion of *C*_60_ on graphene. The motion shows a diffusive behavior which consists of either a continuous motion or discrete movements between trapping sites depending on the type of the graphyne sheet. For graphyne-4 and graphyne-5, fullerenes could detach from the surface of the graphyne sheet at room temperature which was not reported for similar cases on graphene sheets. Collective motion of a group of fullerenes interacting with a graphyne studied and it is shown that fullerenes exhibit stable assemblies. Depending on the type of graphyne, these assemblies can have either single or double layers. The mobility of the assembled structures is also dependent on the type of the graphyne sheet. The observed properties of the motion suggests novel applications for the complexes of fullerene and monolayer graphynes.

Creating a desired self-assembled pattern from random motion of nanoparticles on a given surface, has been one of the important questions in science and technology of manufacturing nanoscale devices, with potential possible applications in various fields. In the last decade, utilizing 2D nanostructures (especially graphene) has proposed possibility of creating 2D patterns by the aid of functionalization, defects and even Moire patterns^1^,^2^,^3^. Among them, graphyne has attracted attention because of its geometry and potential applications. Graphyne is an allotrope of carbon consisting of Carbon-Carbon acetylenic bonds and benzene rings with different ratios which dictate its geometry and type (Fig. 1). Beside its mechanical and thermal properties^4^,^5^,^6^, the nanoporous nature of graphyne with higher diversity of pore size in comparison to graphene, makes it a sound candidate with a higher capacity for atomic scale separation purposes. Studies suggest promising roles for graphyne in membrane filtration and separation technology as well as hydrogen storage, sensors and electronic devices^7^,^8^,^9^,^10^,^11^,^12^,^13^. Here we introduce other interesting aspects of the graphyne sheets which can extend their application to substrates for manipulation and assembly of nanoparticles.

Understanding the phenomena occurring at the nanometric scales, such as manipulation and transportation helps to take advantage of these nanostructures for more novel applications. Self-assembled structures offer development of new devices with variety of applications^14^,^15^,^16^. Ordering and assembly of fullerene particles on various surfaces was one of the dominant topics for researchers of the field of nanotechnology and nanoarchitectonics^17^,^18^,^19^,^20^,^21^,^22^,^23^,^24^,^25^,^26^,^27^,^28^,^29^,^30^. Many studies have highlighted the motion and aggregation of different nanostructured materials such as carbon nanotubes, fullerenes and water droplets on graphene^31^,^32^,^33^,^34^,^35^. The motion of nanoparticles spontaneously forms ordered aggregates which can be controlled with different stimuli such as temperature, electric voltage, or the geometry of the substrate^33^,^36^. Recent study by Leither *et al.* investigates charge transport and conductance properties of the self-assembled supramolecular fullerenes structures which can be used in organic filed-effect transistors (FETs)^37^. Graphyne with various hole sizes provides an intriguing substrate to control the path of particles as charge carriers which are confined on the 2D self-assembled layers. Moreover, graphyne can be suggested as a novel nanostructured substrate for controlling the arrangement of particles and formation of porous nano networks. These patterns can be used for fabrication of scaffolds for different purposes including tissue engineering^38^. In addition to the intermolecular interaction, the interaction between the substrate and moving particles plays the main role in the final assembled structures. Since even subtle alterations of the geometry at the atomic scale cause considerable changes of thermal, electrical, optical, and mechanical properties of the nanostructures, the results of the transportation on other substrates like graphene can not be extended to graphyne. Our study suggests that the unique geometry of graphyne sheets can help to have control over the size, mobility, and number of the layers of fullerene assemblies.

This paper aims to study the interaction of fullerene particles with graphyne monolayers with molecular dynamics (MD) simulations. Study of self-assemblies requires a comprehensive insight into the motion of the individual particles on the substrate. Thus, we study the diffusion of fullerenes on the surface as well as its vertical vibrations between the energy wells. Furthermore, we have analysed the arrangement of clusters to investigate the self-assembly of fullerenes on graphyne sheets with different geometries. Five different types of graphene sheets are chosen for this study. The sheets are schematically represented in Fig. 1. A graphyne sheet consists of hexagonal rings which are connected via acetylenic linkages to each other. The acetylenic linkages form a triangular region with hexagons being located at the corners. The linkage between two hexagonal rings consists of 2, 4, 6, 8, and 10 carbon atoms in graphyne-1 to graphyne-5, respectively. Theoretically, there are infinite types of graphyne depending on the number of carbons in the acetylenic linkage, but they might not be energetically stable. It is worth mentioning that numerous experminetal studies have been dedicated for the last two decades to introduce methods for fabrication of the graphyne. The subunits of some types of graphyne has been already synthesized in laboratories and various methods proposed for synthesis of other similar all-carbon structures and networks^39^,^40^,^41^,^42^,^43^,^44^,^45^. These studies beside the recent success on fabrication of graphdiyne^46^ (a member of graphyne family) demonstrate the possibility of fabrication of other types of graphynes in large scales in the near future.

## Results and Discussion

### Energy landscape of interaction of a single *C*_60_ and graphynes

**Figure 2:**
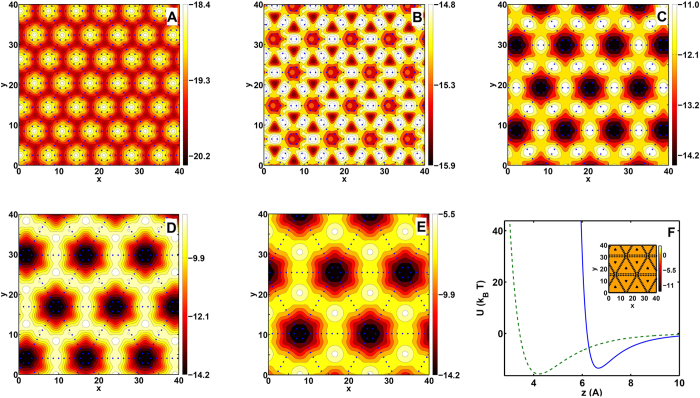
(**A–E**): Landscape of the potential energy felt by a *C*_60_ passing over a graphyne-n with *n* = 1..5 at a height of 6.5 Å (calculated from the center of mass of fullerene), respectively. F: dependence of the potential energy between fullerene and graphyne-5 to distance of the center of mass of fullerene from graphyne at two different positions. dashed green curve corresponds to the center of mass located above center of a triangle and the blue solid curve is for that being located above the center of a hexagon. F(inset): the energy landscape for graphyne-5 at the height of 4.2 Å of its center of mass from graphyne. All the above plots are obtained using cut-off distance of 10 Å between carbon atoms.

The energy landscape of fullerene above a graphyne monolayer could give an intuitive description of the motion. Figure 2 shows these energy landscapes of fullerene on different types of graphyne. The interaction between *C*_60_ atoms and the graphyne atoms is described with a 6–12 Lennard-Jones potential which has been widely used in previous studies on the interaction of carbon nanostructures^31^,^33^,^47^,^48^. We chose 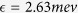 and *σ* = 0.34 *nm*. The fullerene has been placed in a distance of 6.5 Å from its center of mass to the graphyne with one of its pentagons facing the graphyne sheet. The interaction is dependent on the orientation of the fullerene, but it should be noted that altering the orientation of the fullerene do not dramatically change the energy landscapes. Figure 2 shows that for graphyne-1 at this particular height, the positions above the hexagons and the triangles belong to maxima and minima of the energy landscape, respectively. Due to its higher density of carbon atoms compared to other graphynes (*n* > 1), it possesses the highest magnitude of interaction energy with the fullerene. For graphyne-2 interaction of *C*_60_ with the surface becomes weaker and both hexagons and the center of triangles become attractive potential wells. In this case, when fullerene is located on a triangular region, it feels a slightly higher attractive energy. For graphyne-3 to graphyne-5, since the number of the carbon acetylenic linkages increases, the unoccupied triangular regions between them become larger. As a results, the interaction of the fullerene with the sheet reduces at this particular height from the surface (6.5 Å). It can be seen that for these types of graphynes the hexagons become attractive potential wells. On the other hand, for larger values of *n*, the potential energy landscape becomes more inhomogeneous and its magnitude varies over larger intervals. For graphyne-4 and graphyne-5, these variations are about 6.2 *kT* and 8.8 *kT*, which makes it difficult for fullerene to escape from a hexagonal spot, at room temperature. It should be noted that in addition to the position of the fullerene on *xy* plane, these energy landscapes also depend on the distance of the *C*_60_ from the graphyne. For example, locating the center of mass of a fullerene at a distance of 4.2 Å from the graphyne-5 changes the energy landscape as shown in the inset of Fig. 2F. Due to the repulsion between the carbon atoms at this distance, fullerene tends to be located inside the sparse triangular regions between acetylenic linkages where they can interact with more carbon atoms of the surface which are in a proper distance from the fullerene atoms. In the Fig. 2F the dependence of the interaction energy versus distance is presented for graphyne-5 at two positions. The blue solid curve and green dashed curve correspond to the *C*_60_ being located above the center of a hexagon and above a triangle, respectively. The latter has a slightly deeper minimum which occurs at about 2.3 Å closer to the surface even though a comparison of Fig. 2E and inset of Fig. 2F suggests that the former potential well is much wider than the other one.

To sum up, the analysis of the energy landscape reveals that there are two possible types of the attractive potential wells depending on the type of the graphyne sheet and distance between *C*_60_ and graphyne. These absorbing wells become more important for higher *n* values where the attractive energy drops significantly outside these points.

### Diffusion of a *C*_60_ on graphyne sheets

**Figure 3:**
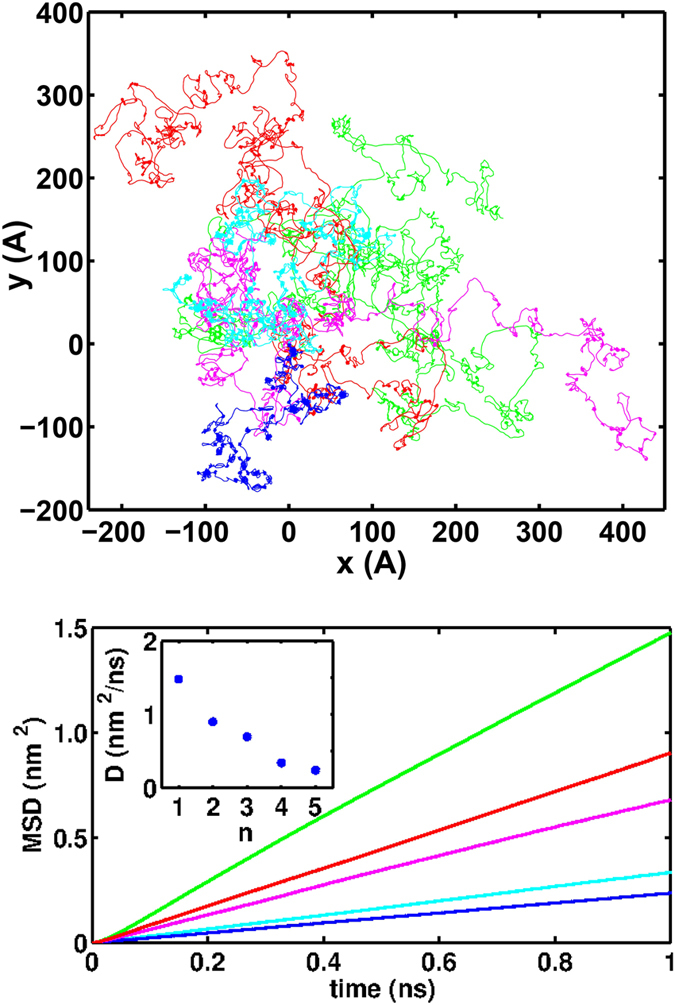
Top panel: Sample trajectories of *C*_60_ on the graphyne-1 (green), graphyne-2 (red), graphyne-3 (pink), graphyne-4 (cyan), and graphyne-5 (blue) in xy plane. This figure shows that as the *n* grows, the mobility of fullerene on graphynes reduces. Bottom panel: Corresponding mean square displacements averaged over 8 realizations. The colors are the same as the top panel. Inset: values of diffusion coefficients for motion of fullerene on different types of graphyne.

Figure 3A shows sample trajectories of the motion of *C*_60_ on different types of graphyne. It qualitatively shows that as *n* increases, the energy landscape felt by *C*_60_ would possess separate deep potential wells which can trap *C*_60_ and decrease its mobility. The mean square displacement (MSD) of the center of mass of the fullerene in the two dimensional (*xy*) plane can be used to quantify the mobility. In the bottom panel of Fig. 3, the MSDs are plotted for different graphynes. To obtain smoothe curves, these plots are obtained by averaging over eight realizations (repeating simulations with different initial conditions) and the results were also averaged over time. At first glance, it can be seen that the obtained values for the MSDs are about an order of magnitude smaller than the similar values reported for the motion of *C*_60_ on graphene^31^,^33^. Secondly, the MSD reduces by increasing *n*. To determine the regimes of the motion in each case, a power law curve was fitted to MSD curves. Since the averaged calculated powers were very close to unity, within the errorbars, we used linear regressions to the first nanosecond of the MSD plots and extracted the diffusion constants. The results are shown in the inset of Fig. 3. The diffusion constants decrease as the number of the acetylenic linkage increases.

**Figure 4:**
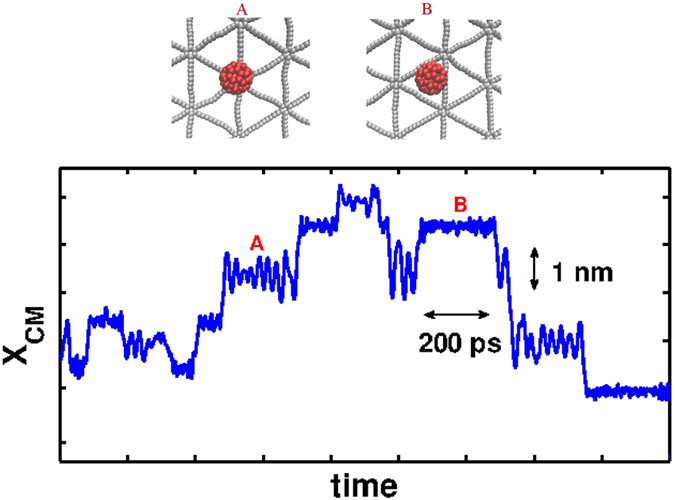
Top: Schematic representation of the two possible trapping sites in a graphyne-5. Bottom: Sample of the x component of the trajectory of the center of mass of a fullerene on graphyne-5. The plot shows the difference in vibrational frequencies at the two types of the trapping sites.

In Fig. 4, *x* component of fullerene coordinate on graphyne–5 is plotted versus time. In this figure, it is easier to recognize the trapping sites at the different potential wells. In the trapping sites, fullerene vibrates until it escapes from the potential well. As it was discussed in the previous section, there are two possible absorbing points: above a hexagon or above a triangle (type A and type B in Fig. 4). Since the vibrational frequency of the trapped fullerene in A and B states are different, Fig. 4 contains the information to recognize in which type of absorbing points the fullerene exists. This figure shows that in the case of graphyne-5 the vibrational frequencies are higher for the trapping triangles (state B) which is due to their small width (see inset of Fig. 2F). By plotting the same curves for the motion of fullerene on all types of studied graphyne sheets, and monitoring the fullerenes in trapped states, it is possible to measure the characteristic period, *τ*_*p*_, of the vibrations at the trapping sites (Table 1). Graphyne-1, graphyne-2 and graphyne-3, just exhibit one type of traps which is the triangular regions between the acetylenic linkages and the period of the vibration reduces as *n* increases. This reducing behavior is still observed for graphyne-4 and graphyne-5, but second trapping states occur in front of hexagons. The latter sites has vibrational periods of about three times higher than the other potential wells. Sample movies of the trapping events of the motion are available in Supporting information. The movies also show preference of fullerene sliding mechanisms against rolling to move during the process of escape from the wells.

**Figure 5:**
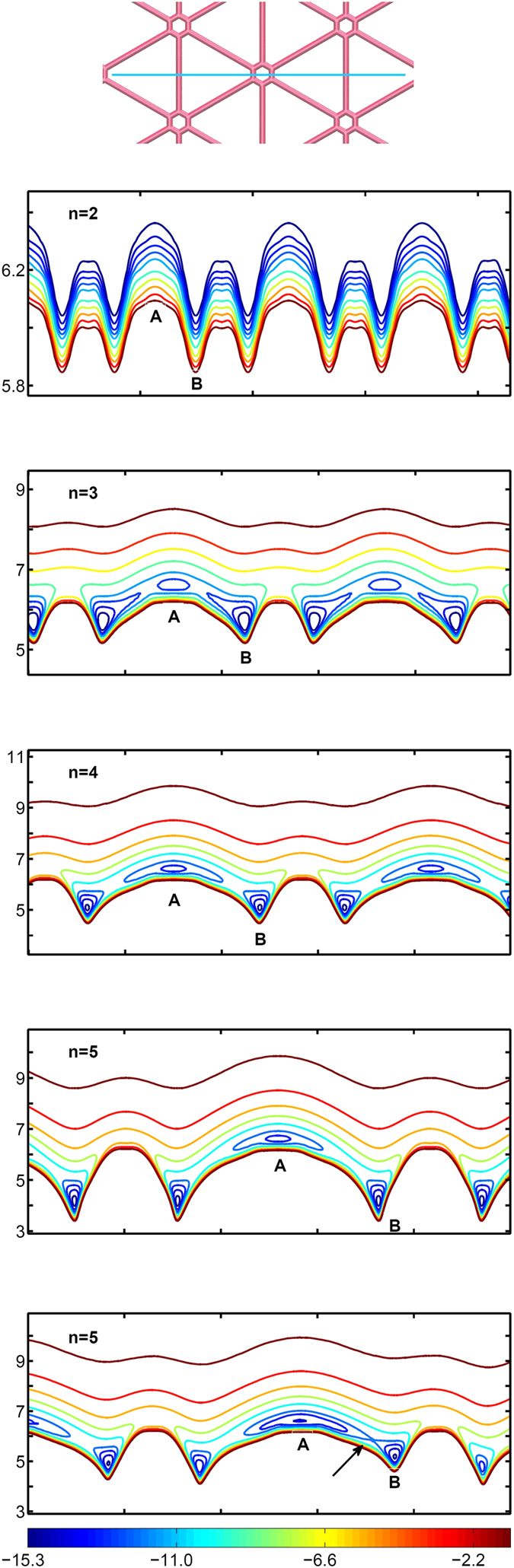
Energy landscape of a *C*_60_ for different heights while moving along a pathway shown in (**a**) from center of triangles, center of the hexagon, and over the acetylenic linkage for different graphynes (**b**–**e**). (**f**) Same energy landscape for a sample graphyne 5 with fluctuations in the two dimensional space. Such fluctuations can ease the transitions between A and B potential wells like the one shown with the arrow in the figure. The markers on the x-axis are 10 Å apart.

A closer look at the motion of *C*_60_, reveals a fairly smooth and continuous motion on graphyne-1 and graphyne-2 while for *n* = 4 and 5 the motion of fullerene can be divided to discrete transitions between the A and B absorbing sites (Supplementary movies). Interestingly, these transitions are not equally weighted. In fact the most frequent transition appears to be between neighboring A and B trapping sites and transitions from A to A or B to B rarely happen. To understand the differences between these transitions, we have measured the vertical dependency of energy landscape on a specific straight fullerene pathway along the center of a triangle and center of a hexagon for all studied graphynes (Fig. 5). It can be seen that for smaller *n* values, the energy landscape is much smoother with respect to the height of fullerene. Furthermore, energy minima at type A and type B trapping sites occur almost at the same level of height and energy. By increasing *n*, the heights which minima were observed at A and B become more distinct. For graphyne-5, this difference is about 2.3 Å which indicates the same feature that was shown in Fig. 2 that for *n* = 4, 5 the center of hexagons and triangles are distinct islands which can trap *C*_60_. According to Fig. 5, for *n* = 4, 5 the potential well corresponding to trap A is wider than trap B which makes it more difficult for fullerene to escape from A than B (See also supplementary movies for the motion of *C*_60_ on graphyne-4 and graphyne-5). According to these plotted energy landscapes, one can see that the energy barrier for B to B transitions is higher than A to B and the difference between these transitions becomes more distinct as *n* grows. Moreover, since the shortest distance between two A traps are longer than the distance between A–B neighbor traps, thermal fluctuations could stimulate A to B transitions. To observe the effect of thermal fluctuations on this energy landscape, we created a two dimensionally distorted graphyne sheet by taking one snapshot of the equilibrated graphyne-5 and projecting its coordinates on the two dimensional space. The results of the same analysis for a part of this sheet is shown at the bottom panel of the Fig. 5. This figure illustrates how thermal fluctuations helps the A to B transition by creating a pathway between A and B.

### Vertical vibration and stability of *C*_60_ on graphyne

In addition to the in-plane motion of the fullerene, it has an effective vibrational motion in normal direction (*z* direction). The thermal energy of the fluctuations in *z* direction can be written as *U*_*z*_ = *k*_*B*_*Tp*(*z*), where *p*(*z*) is the probability distribution function of *z* component of the motion of the center of mass of fullerene. Fitting parabolic functions to this energy gives an effective vibrational stiffness, *k*_*z*_, for the motion of fullerene in each case. The vibrational stiffness reduces by adding more acetylenic linkages (Table 1). Also all of the values are smaller than the corresponding value for graphene^31^, in agreement with the predictions for the elastic moduli of the graphyne sheets^49^. For graphynes with more number of acetylenic linkaes, the elastic moduli is lower and it makes it easier to bend the sheet. These vertical motions have an important role in the stability of the fullerene on the graphyne sheets. For example, by repeating the same simulation for the *C*_60_ on graphyne-5 with different initial conditions, in some cases, *C*_60_ desorbs from the surface. For graphene case at room temperature^31^,^33^ due to the strong interaction between fullerne and graphene this feature could not be observed. The fullerene detaches from the surface with a linear velocity in range of 50–200 *m*/*s* and angular velocity of 55–70 *rounds*/*ns*. All of the observed detachments occur from type A (See Fig. 4). After leaving a well, the fullerene has enough momentum to escape from the graphyne surface. Having lower depths and being located in a bit lower distances with respect to the surface, makes it harder to escape from the sheet from type B wells. The probability of the escape depends on the temperature. For instance the process is observed more frequently in our simulations for graphyne-5 at the elevated temperatures of about 400 K and the escape is not observed at temperatures below 200 *K* during the 30 ns simulations (results not shown). beside graphyne-5, graphyne-4 is the only one which fullerene could escape from the surface binding at room temperature, even though this process rarely happens. For the other types of graphyne the escape have not been observed during the time scale of 30 ns. According to the short time scale of the simulations, one can conclude that for graphynes 4 and 5, it is not possible to deposit single *C*_60_s on the surface at room temperature because after a very short time from deposition, *C*_60_ particle will leave the surface.

### Self-assembly of fullerenes on graphynes

**Figure 6:**
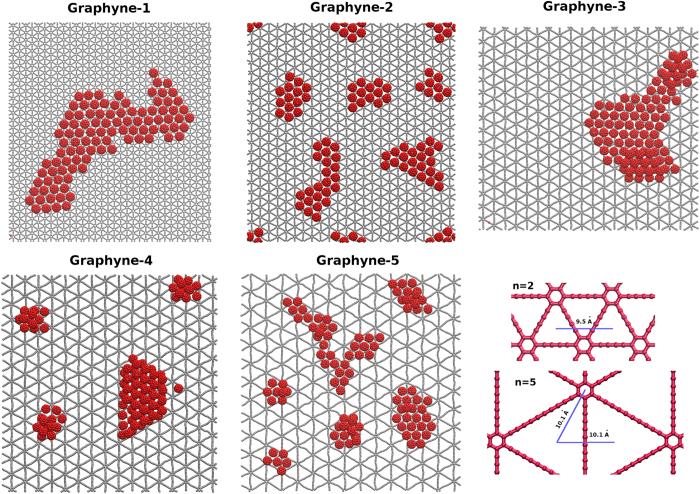
Snapshots of the simulation of *C*_60_ assemblies on the graphyne-1 to graphyne-5. The lower right image schematically shows the distance between the potential wells in graphyne-2 and graphyne-5 which tend to make stable and less mobile assemblies. See the main text for more information.

We carried out further simulations on groups of fullerenes to see how they assemble on a graphyne sheet. We studied assemblies of 100 *C*_60_ particles on the surface of each of the 5 graphynes (Fig. 6) during a 10 ns simulation. The equilibrium center to center distance for van der Waals interaction of two interacting fullerenes is approximately 10 Å^50^. During the simulation, fullerenes tend to make assemblies with the 10 Å distance in between. In the case of graphyne-1 this process occurs quickly and after a few nanoseconds all of the fullerenes stick together, creating a single layer assembly. According to Fig. 2, the attractive van der Waals energy between fullerene and the surface of the graphyne-1 is very high which leads to the fact that the assembled fullerenes tend to make a single layer on it to minimize the potential energy. In very rare cases we observe a *C*_60_ jump on the others but after a while that particle returns to the surface beside other fullerene particles. This assembly can still move on the surface of graphyne-1 but as it becomes larger, it becomes less mobile.

For graphyne-2 distance between the center of two triangular potential wells is 4.8 Å which makes the distance between the center of two non-adjacent triangles 9.6 Å, very close to the equilibrium distance of *C*_60_-*C*_60_ interaction (see Fig. 6 lower right panel). This means that fullerene particles can stick together and at the same time remain at their minimum potential energy positions with respect to the graphyne-2 surface. As a result, they produce stable single layer islands which hardly move on the surface in the time-scale of our MD simulations.

For graphyne-3 the nearest distance between A and B sites is 7.3 Å. However, fullerenes still have a fairly suitable interaction with the surface and after a few nanoseconds small clusters of fullerenes can find each other to create a large assembled structure (see supporting movies). Fullerenes prefer to stay together rather than be located at the graphyne trapping sites. Accordingly, on this graphyne sheet the structure mostly consists of a single layer of assembled fullerenes even though at some parts a double layer of fullerenes appears. Since there is a mismatch between the equilibrium distance of fullerenes and the distance between the graphyne-3 minimum potential sites, these assemblies are more mobile than those on graphyne-2 (see supporting movie).

In graphyne-4, the hopping between A and B sites leads to have a more discontinuous diffusive motion rather than what we have for the case of graphene or graphyne with *n* = 1,.. 3. The separate A and B sites become absorbing potential wells which can trap and hold *C*_60_. On the other hand, the distance between the nearest A and B sites is 8.9 Å which does not match the lattice constant of *C*_60_ assembly. As a result, some fullerenes jump to the top of the others and create a two-layer structure to compensate the energy cost due to this mismatch. This interesting property distinguishes *C*_60_ assemblies on graphyne from their predicted structure on graphite. According to a computational study, in the latter case double layers are only possible for a large number of particles otherwise the edge effects will push the structure toward single layers^51^. During the simulation on graphyne-4, 3 fullerenes left the surface of the graphyne which occurs at early stage of the simulation and the particles become stable on the surface after creation of the assemblies.

In graphyne-5, the distance between the type A and B traps is 10.1 Å which is again very close to fullerenes equilibrium distance. Similar to the case of graphyne-2, fullerenes generate stable structures in separate islands but this time by sitting on the A and B sites while they are located at their own equilibrium distance. In this case no escaping from the graphyne sheet were observed which indicates that unlike the single fullerenes, it’s possible to have stable *C*_60_ monolayer assemblies on this type of graphyne.

Our results suggest a simple way to make fullerene assemblies in one or two layers or making mobile or immobile and stable assemblies depending on the type of the sub-layer graphyne.

## Conclusion

We found new features in diffusion and self-assembly of *C*_60_ on graphynes which extends their possible applications in the future. Beside the interesting physical properties of a fullerene motion on a graphyne sheet, the obtained results suggest new opportunities to develop devices based on the fullerene and graphyne complexes. Using different types of graphyne, one can control the mobility of fullerene or other particles on the sheets. But as previously mentioned, the graphene sheets remain better options if a higher mobility is desired. The other unique property of the motion on graphyne which distinguishes it from the graphene is the ability of escaping from the surface of the sheet which is observed for graphyne-5 and graphyne-4. To deposit and stabilize particles on the surface of these sheets, one can either reduce the temperature or use assemblies instead of single particles. It was also highlighted that different types of self-assemblies can be obtained depending on the *n* value. Some graphyne sheets including graphyne-1, produce mobile assemblies, while graphyne-2 and graphyne-5 make stable assemblies with a very small mobility. For graphyne-4 difference between the distance of the potential wells on the surface and the equilibrium distance of two fullerenes results in the creation of double layer fullerene assemblies. By using different types of graphyne, one can also have control over the creation of one-layer or two-layer *C*_60_ assemblies. Double layer assemblies offer the possibility of fabrication of 3D networks and scaffolds which is one the biggest challenges in tissue engineering^38^. It is worth mentioning that the successes on synthesis of graphyne-1 and graphyne-2^46^,^52^,^53^,^54^, allows to experimentally test the obtained results for mobility and self-assemblies on these two types of graphyne. All of these stunning properties of the motion and self-assemblies studied in this paper, provides an abundance of opportunities for their application in the field of nanotechnology research.

## Method

Molecular dynamics (MD) have been carried out by Large-scale Atomic/Molecular Massively Parallel Simulator (LAMMPS) software^55^. Different graphyne structures with the approximate size of 20 × 20 *nm*^2^ created in a box with the periodic boundary conditions in graphyne plane. The adaptive intermolecular reactive empirical bond order (AIREBO) potential^56^ is used to define interaction of carbon atoms. This interaction potential have been used previously to study mechanical and thermal properties of graphyne^4^,^5^,^6^. The Lennard-Jones cutoff between all carbon atoms for AIREBO potential considered to be 8.5 Å. The structures of the graphynes are created based on ref. 57. After minimization of the structures with the AIREBO potential, we locate the center of mass of the *C*_60_ on a height of 6.8 Å from graphyne. We start with 5000 steps of minimization and then perform a NPT simulation for 5 *ns* at 300 *K* to obtain optimized structure of the graphyne sheet. Then the system is simulated in NVT ensemble using Nose-Hoover thermostat at 300 K for 25 ns and the position of the fullerene has been sampled to obtain characteristic properties of the motion of the fullerene on each graphyne sheet. For self-assembly studies we use the same protocol but this time we use a group of 100 fullerenes which are initially located on an ordered simple cubic lattice above the graphyne sheet and the simulation were performed for 10 ns. Timestep in all of our simulations is one femtosecond. All of the figures of the atomic structures have been created by VMD^58^.

## Additional Information

**How to cite this article**: Ozmaian, M. *et al.* Diffusion and self-assembly of *C*_60_ molecules on monolayer graphyne sheets. *Sci. Rep.* **6**, 21910; doi: 10.1038/srep21910 (2016).

## Supplementary Material

Supplementary video 1

Supplementary video 2

Supplementary video 3

Supplementary video 4

Supplementary video 5

Supplementary video 6

Supplementary video 7

Supplementary video 8

Supplementary video 9

Supplementary video 10

Supplementary video 11

Supplementary Information

## Figures and Tables

**Figure 1:**
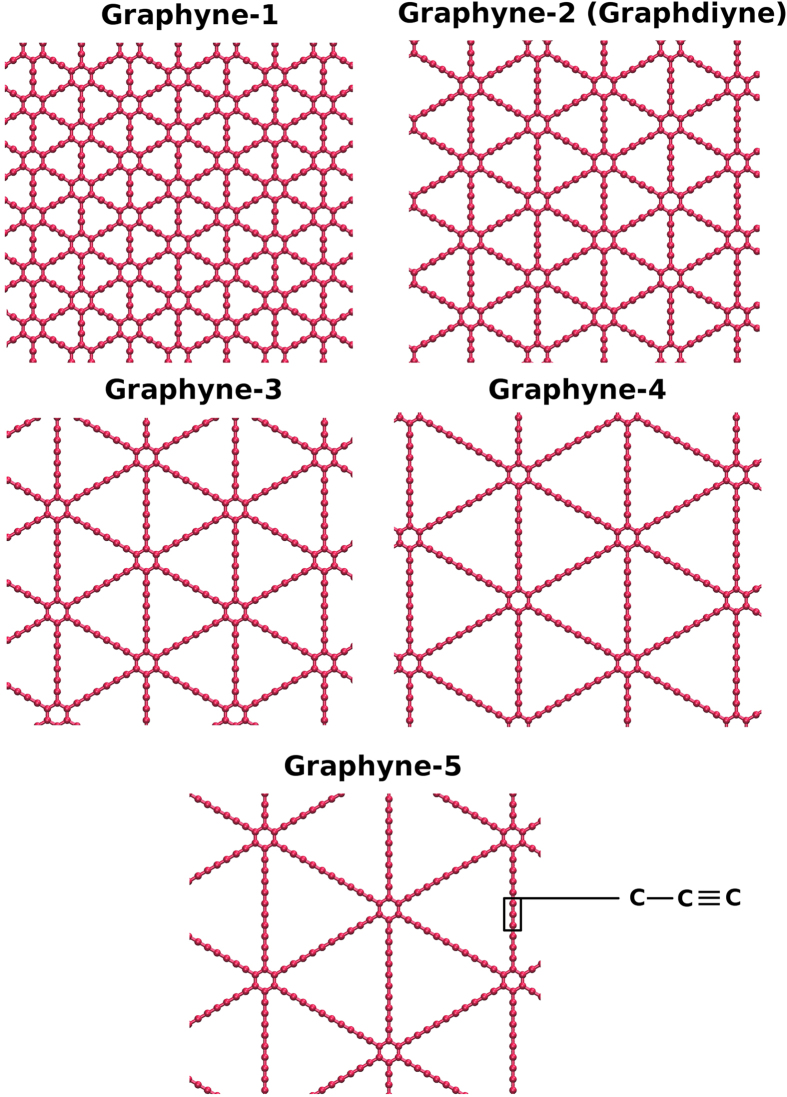
Different types of graphyne sheets studied in this paper. Each graphyne is named with an index “*n*” which indicates the number of carbon-carbon triple bonds in a link between two adjacent hexagons.

**Table 1:** Some characteristic properties of the motion of fullerene on graphyne-n.

n	*k*_*z*_ (kT/Å^2^)	*τ*_*p*_(*ps*)
1	0.77	16.5 ± 2.0
2	0.71	12.5 ± 1.5
3	0.36	10.5 ± 1.0
4	0.28	9.0 ± 1.5 (24 ± 3.0)
5	0.22	8.5 ± 1.0 (23 ± 3.0)
graphene^31^	1.25	—

*τ*_*p*_ values in the parentheses belong to the A trapping sites.
