# Supplementary material for: Diffusion and self-assembly of C60 molecules on monolayer graphyne sheets
Source: Sci Rep. 2016 Feb 25;6:21910. doi: 10.1038/srep21910 (PMC4766508; doi:10.1038/srep21910)
Supplement: Supplementary Information [file srep21910-s12.doc]

**Supporting information**

**Diffusion and self-assembly of C60 molecules on monolayer graphyne sheets**

*M. Ozmaian, A. Fathizadeh, M. Jalalvand, M. R. Ejtehadi, SMV Allaei*

**Description of movies:**

Sample movies from the motion of single or assembly of fullerenes are available via supporting information. Movies 1 to 5 correspond to the motion of single fullerene particles on graphyne-1 to graphyne-5, respectively. Movie 6 shows one of the escaping events that a fullerene leaves the surface of a graphyne-5 from a type A potential well. movies 7 to 11 show the simulation of self-assemblies of 100 fullerenes on graphyne-1 to graphyne-5.

**Video Legends:**

Movie1: Sample simulation of fullerene on Graphyne-1

Movie2: Sample simulation of fullerene on Graphyne-2

Movie3: Sample simulation of fullerene on Graphyne-3

Movie4: Sample simulation of fullerene on Graphyne-4

Movie5: Sample simulation of fullerene on Graphyne-5

Movie6: Sample of detachment events in simulation of fullerene on Graphyne-5

Movie7: Sample simulation of fullerene assemblies on Graphyne-1

Movie8: Sample simulation of fullerene assemblies on Graphyne-2

Movie9: Sample simulation of fullerene assemblies on Graphyne-3

Movie10: Sample simulation of fullerene assemblies on Graphyne-4

Movie11: Sample simulation of fullerene assemblies on Graphyne-5
